# Association between inflammatory bowel disease and risk of stroke: a systematic review and meta-analysis of cohort studies

**DOI:** 10.3389/fneur.2023.1204727

**Published:** 2023-11-17

**Authors:** Jin-Shan Fan, Meng Wang, Ni Chen, Bai-chao Sun, Qi-Bing Zhang, Yong Li, Ming-Jie Huang

**Affiliations:** ^1^Department of Intensive Care Unit (ICU), Qian Jiang Central Hospital of Hubei Province, Qian Jiang Hospital Affiliated to Renmin Hospital of Wuhan University, Qian Jiang Clinical Medical College, Health Science Center, Yangtze University, Qianjiang, China; ^2^Department of Neurology, The Third Clinical Medical College of China, Three Gorges University, Gezhouba Central Hospital of Sinopharm, Yichang, China; ^3^Department of Ophthalmology, The Third Clinical Medical College of China, Three Gorges University, Gezhouba Central Hospital of Sinopharm, Yichang, China

**Keywords:** IBD, stroke, risk, meta-analysis, systematic review

## Abstract

**Background/objectives:**

Recently, four meta-analyses have explored the association between inflammatory bowel disease (IBD) and the risk of stroke. These studies have demonstrated that people with IBD may be at an increased risk of stroke. However, some limitations such as high heterogeneity and the lack of uniformity in the types of research, especially the reuse of some sample sizes, cannot be neglected. These factors reduce the credibility of their research conclusions. Therefore, we conducted a meta-analysis to explore this possible association.

**Methods:**

PubMed, Embase, and Web of Science were searched from inception to 30 June 2023. A random effects model with the generic inverse variance method was used in this meta-analysis. The Review Manager software was used to obtain all relative risks (RRs) and their 95% confidence intervals (CIs). Publication bias was tested, and sensitivity and subgroup analyses were conducted to explore possible heterogeneities.

**Results:**

This meta-analysis included 12 cohort studies (involving 4,495,055 individuals). Meta-analysis of these data has shown that IBD was associated with an increased risk of stroke (RR = 1.19, 95%CI:1.14-1.24, p < 0.00001). Our results were stable and robust in subgroup and sensitivity analyses.

**Conclusions:**

Our results suggest that IBD is associated with an increased risk of stroke. To reduce the incidence of stroke, patients with IBD are encouraged to undergo stroke risk assessments, especially for young female patients; assessing the risk of ischemic stroke is of particular importance. Prospective studies considering stroke subtypes, IBD severity and treatments, regions, and other confounding factors are needed to further explore the nature of each association.

**Systematic review registration:**

https://www.crd.york.ac.uk/PROSPERO/, identifier CRD42022373656.

## 1 Introduction

Inflammatory bowel disease (IBD) is a chronic inflammatory disease in the gastrointestinal tract, with Crohn's disease (CD) and ulcerative colitis (UC) being types of the condition. The epidemiology of IBD has changed significantly compared to the past. The highest rate of inflammatory bowel disease (IBD) is observed in the Western world, yet in newly industrialized countries in Asia, Africa, and Latin America, the incidence of IBD is increasing at a rapid rate (1–3). With its continual growth on a global scale, IBD has become an immense economic strain on health systems and a major healthcare issue across the world (4).

According to the Global Burden of Disease 2019, stroke has become the second leading cause of death and the third leading cause of disability and death combined globally. From 1990 to 2019, the total number of stroke events rose by 70.0%, epidemic stroke increased by 85.0%, and stroke deaths increased by 43.0% (5). The World Stroke Report indicates that in 2019, more than 120 million people died from stroke and related diseases (6, 7), resulting in a huge economic burden and psychological distress for families and society (8). Furthermore, according to some studies, it has been estimated that ~2 million adolescents suffer from ischemic strokes annually, which makes up 15–18% of all ischemic strokes globally, with the stroke rate among the younger generation also increasing (9–12). It is yet to be determined precisely what causes stroke, however, some studies have demonstrated that an immune inflammatory response can be detrimental to the pathophysiology of stroke (13–15).

Studies have revealed a close association between IBD and a range of neurological disorders, including Parkinson's disease, dementia, multiple sclerosis, and depression (16–19). Given the growing prevalence of stroke, it is worth investigating whether IBD could be a potential risk factor for stroke. Previously, four meta-analyses have found that IBD is a risk factor for stroke (20–23). Although these studies have reached consistent conclusions, we also found that these studies have high heterogeneity, a small or incomplete number of included articles, and a lack of uniformity in the types of research. These reasons led to the conclusion that these articles should be treated with caution. Therefore, to establish whether IBD is a contributing factor for stroke, we conducted a systematic review and meta-analysis by scrutinizing all the cohort studies in the relevant databases and analyzing the data collected.

## 2 Methods

### 2.1 Protocol and registration

We performed the systematic review and meta-analysis in accordance with the PRISMA (the Preferred Reporting Items for Systematic Reviews and Meta-Analysis) (24) reporting guidelines. This review protocol was prospectively registered on the International Prospective Register of Systematic Reviews (PROSPERO Registration Number: CRD42022312797).

### 2.2 Search strategy

We conducted a comprehensive search of PubMed, Embase, and Web of Science from inception to 30 June 2023 to identify all studies related to the association between inflammatory bowel disease (IBD) and stroke. The following keywords were used in our search strategies: (inflammatory bowel disease OR ulcerative colitis OR Crohn's disease) AND (stroke OR cerebrovascular disorder OR cerebral infarction OR cerebral hemorrhage). We conducted our search combining MeSH terms with text word searching, without any language restriction. The online Supplementary material (Supplementary Table 1) presents the details of the retrieval strategy for PubMed. In addition, we manually searched the reference lists of all relevant articles to ensure completeness.

### 2.3 Study selection

The included studies met the following inclusion criteria: (1) cohort studies; (2) the exposure was IBD and the outcome was stroke appearing after the diagnosis of IBD; and (3) studies reported relative risk (RR), hazard ratio (HR), or incidence rate ratio (IRR) with 95% confidence interval (CI) for stroke incidence or provided the original data for the calculation. The exclusion criteria were as follows: (1) laboratory studies, abstracts, reviews, meta-analyses, comments, letters, and case reports; (2) articles without sufficient data; (3) IBD appearing after the diagnosis of stroke; and (4) the study did not provide sufficient data to calculate the RR, HR or IRR and 95% CI. Studies were independently screened by two reviewers who read titles and abstracts and obtained the full text of potentially relevant articles based on the inclusion and exclusion criteria. Any differences were resolved by consensus.

### 2.4 Data extraction

We extracted the following information from each eligible study: the first author, the year of publication, districts, study subjects, sample size, follow-up duration, type of IBD and stroke, mean age or age of the group, sex, the proportion of males, and RR or HR (adjusted and unadjusted) with their 95%CI. Discrepancies were settled with mutual agreement by two reviewers who independently extracted the data from each included study.

### 2.5 Assessment of quality

The Newcastle-Ottawa Scale (NOS) (25), which is specially designed for observational and non-randomized studies, was used to evaluate the quality of the included articles. It uses a star system (ranging from 0 to 9 stars) to assess the quality of a study based on three domains: selection (four stars), comparability (two stars), and outcome/exposure (three stars). The number of stars signifies the quality of an article: 7–9 stars being high-quality, 4–6 stars being moderate quality, and 1–3 stars being low-quality.

### 2.6 Statistical analysis

This study aimed to investigate the association between IBD and the occurrence of ischemic stroke, hemorrhagic stroke, and unclassified stroke. Meta-analyses were performed using the Review Manager 5.3 (Cochrane Collaboration, Copenhagen, Denmark). RR and 95% CI from each study were calculated using a random-effects model, the generic inverse variance method of DerSimonian and Laird. We used Cochran's Q-test and I^2^ statistic to evaluate heterogeneity among studies. Cochran's Q-test was used to evaluate the heterogeneity, *P* < 0.10 for the Q-test was considered statistically significant and I^2^ value was used to assess the degree of heterogeneity. If I^2^ values were <25%, we considered low heterogeneity, values in the range of 26%-50% were considered as having medium heterogeneity, and above 50% as having high heterogeneity. When the heterogeneity was >50%, it was necessary to employ a random effects model to combine the data; otherwise, a fixed effects model was used. Sensitivity/subgroup analyses were performed according to the type of IBD and stroke, race, age, and sex of study participants, follow-up period, and adjustment for confounders to explore the potential sources of heterogeneity. Additionally, the funnel plot and Egger's regression asymmetry were used to evaluate the potential of publication bias with the STATA/SE software (Version 12.0, STATA Corporation, Texas, USA).

## 3 Results

### 3.1 Selection

As specified in the PRISMA flow diagram, there were 3,431 potentially relevant articles retrieved from the three electronic databases using the initial search strategy. A total of 1,269 duplicates were excluded. By reading the titles and abstracts, we obtained 68 records, of which 12 articles were finally included after reading the full texts (Supplementary Figure 1).

### 3.2 Study characteristics

A total of 12 cohort studies (26–37) met our inclusion criteria; the main characteristics of the included studies were listed in Table 1. The meta-analysis involved a total of 4,495,055 individuals. The sample size among the included studies varied widely, from 8,060 participants (28) in a Canadian study to 455,950 participants (26) in the United Kingdom Biobank study. The average age ranged from 36.51 to 56.7 years, and the proportion of males ranged from 41.5% to 61%, except for one study (37) that did not mention the male-to-female ratio. The follow-up period also varied from 1 to 16.6 years. Two of the articles (27, 29) did not classify IBD as CD or UC. While only four articles (28, 31, 35, 37) defined the type of stroke. The studies were conducted in different regions, with eight studies (26, 27, 29, 32, 33, 35–39) being conducted in Europe, one study in North America (28), and three studies in Asia (30, 31, 34, 40, 41). According to the quality evaluation of the Newcastle-Ottawa Scale (NOS) results, three articles were of medium quality and nine articles were of high quality (see Supplementary Table 4).

**Table 1:** Main characteristics of included studies.

**References**	**Region**	**Study design**	**Follow-up duration (years)**	**Type of IBD**	**Type of stroke**	**Sample size**	**Mean age or age group (years), male (%)**
Alayo et al. (26)	UK	Cohort	12.4	CD/UC	Unspecified	455,950	56.7 (45.4)
Baean-Diez et al. (27)	Spain	Cohort	6	IBD	Unspecified	9,544	56 (44%)
Bernstein et al. (28)	Canada	Cohort	16.1-16.6	CD/UC	Subarachnoid-hemorrhage, intracranial hemorrhage, ischemic stroke	8,060	CD:36.51 (41.5%) UC:42.42 (48.7%)
Choi et al. (30)	Republic of Korea	Cohort	4.9	CD/UC	Unspecified	37,696	39.4 (61.0%)
Tanislav et al. (36)	Germany	Cohort	1-5	CD/UC	Unspecified	23,894	47 (45.5%)
Huang et al. (31)	Taiwan	Cohort	7	CD/UC	Ischemic stroke	18,392	44.8 (45.1%)
Kirchgesner et al. (32)	France	Cohort	3.5	CD/UC	Unspecified	210,162	45 (46.2%)
Kwon (34)	Republic of Korea	Cohort	8-11	CD/UC	Unspecified	2,746,988	55.65 (61.31%)
Sun et al. (35)	Sweden	Cohort	12	CD/UC	Ischemic and hemorrhagic stroke	491,993	43 (48.57%)
Kristensen et al. (33)	Denmark	Cohort	6.8	CD/UC	Unspecified	260,774	43 (46.5%)
Card et al. (29)	UK	Cohort	5.9	IBD	Unspecified	185,587	45 (47.7%)
Zöller et al. (37)	Sweden	Cohort	4	CD/UC	Ischemic and hemorrhagic stroke	43,832	NR

### 3.3 Association between IBD and risk of stroke

In all, 12 studies confirmed the link between IBD and stroke risk. A pooled analysis showed that IBD was significantly associated with an increased stroke risk (*n* = 17, RR = 1.19, 95%CI:1.14–1.24, *p* < 0.00001 Supplementary Figure 2). Due to the relatively high heterogeneity (I^2^ = 59%, *p* = 0.001, see Supplementary Figure 2), to further explore the potential sources of heterogeneity, we performed several subgroup analyses. Details of all subgroup analyses are provided (see Table 2 and Supplementary Figures 3–9). In a subgroup analysis stratified by type of IBD, we found that stroke was positively associated with the risk of CD (*n* = 11, RR = 1.30, 95%CI:1.20–1.41, *p* < 0.00001; I^2^ = 65% Supplementary Figure 3), UC(*n* = 11, RR = 1.11, 95%CI:1.06–1.16, *p* < 0.00001; I^2^ = 36% Supplementary Figure 3) and any IBD subgroups (*n* = 9, RR = 1.18, 95%CI:1.14–1.21, *p* < 0.00001; I^2^ = 0% Supplementary Figure 3). In subgroup analysis stratified by the follow-up period, we found significant positive association between IBD and stroke risk for patients with a follow-up period of less than 5 years (*n* = 8, RR = 1.21, 95%CI:1.17–1.24, *p* < 0.00001; I^2^ = 73% Supplementary Figure 4) and more than or equal to 5 years (*n* = 9, RR = 1.14, 95%CI:1.10–1.17, *p* < 0.00001; I^2^ = 0% Supplementary Figure 4). It is well known that age, sex, and race are also risk factors for stroke, so we performed subgroup analyses of patients stratified by these variables. In subgroup analysis stratified by age, we found a significant positive association between IBD and risk of stroke with the following results: those aged more than or equal to 40 years (*n* = 12, RR = 1.22, 95%CI:1.10–1.35, *p* = 0.0001; I^2^ = 80% Supplementary Figure 5) and those aged under 40 years old (*n* = 8, RR = 1.46, 95%CI:1.17–1.82, *p* = 0.0007; I^2^ = 63% Supplementary Figure 5). In subgroup analysis stratified by sex, we found a significant positive association between IBD and risk of stroke with the following results: females (*n* = 10, RR = 1.30, 95%CI:1.18–1.42, *p* < 0.00001; I^2^ = 68% Supplementary Figure 6) and males (*n* = 10, RR = 1.12, 95%CI:1.06–1.19, *p* < 0.0001; I^2^ = 31% Supplementary Figure 6). In the subgro up analysis by race, we found a significant positive association between IBD and stroke risk in Caucasians (*n* = 12, RR = 1.21, 95%CI:1.16–1.27, *p* < 0.00001; I^2^ = 64% Supplementary Figure 7) and Asians (*n* = 5, RR = 1.10, 95%CI:1.02–1.19, *p* = 0.02; I^2^ = 17% Supplementary Figure 7). In the subgroup analysis stratified by adjustment for confounders, we found a significant positive association between IBD and stroke with adjusted RR (*n* = 15, aRR = 1.17, 95%CI:1.14–1.19, p <0.00001; I^2^ = 63% Supplementary Figure 8) and crude RR (*n* = 4, cRR = 2.69, 95%CI:2.61–2.78, *p* < 0.00001; I^2^ = 99% Supplementary Figure 8). When exploring the relationship between IBD subtypes and stroke subtypes, we found that CD and UC were associated with the incidence of ischemic stroke (CD *n* = 2, RR = 1.23, 95%CI:1.14–1.32, *p* < 0.00001; I^2^ = 22%; UC *n* = 2, RR = 1.14, 95%CI:1.03–1.27, *p* = 0.009; I^2^ = 76% Supplementary Figure 9), but they were not associated with hemorrhagic stroke (CD *n* = 2, RR = 1.38, 95%CI:0.83–2.30, *p* = 0.21; I^2^ = 93%; UC *n* = 2, RR = 1.16, 95%CI:0.85–1.58, *p* = 0.35; I^2^ = 87% Supplementary Figure 9). No evidence of publication bias was found in our analysis of the association between IBD and the risk of stroke; the visual inspection of the funnel plot and the Egger's test (P = 0.119 Supplementary Figures 10, 11) both provided support for this conclusion. Moreover, no significant changes were observed in the direction and magnitude of the pooled estimates after excluding studies one by one from the meta-analysis. This suggests that the meta-analysis results are reliable and stable (Supplementary Table 5, Supplementary Figure 12).

**Table 2:** Subgroup analyses of the association between IBD and risk of stroke.

**Subgroup**	**No. of studies**	**RR (95%CI)**	**P_association_**	**I^2^ (%)**	**P_heterogeneity_**
**Overall studies**	17	1.19 (1.14–1.24)	P <0.00001	59%	P = 0.001
**Type of IBD**					
Any IBD	9	1.18 (1.14–1.21)	P <0.00001	0%	P = 0.83
CD	11	1.30 (1.20–1.41)	P <0.00001	65%	P = 0.002
UC	11	1.11 (1.06–1.16)	P <0.00001	36%	P = 0.11
**Follow-up period**					
Less than 5 years	8	1.21 (1.17–1.24)	P <0.00001	73%	P = 0.0005
More than or equal to 5 years	9	1.14 (1.10–1.17)	P <0.00001	0%	P = 0.72
**Adjustment for confounders**					
cRR	4	2.69 (2.61–2.78)	P <0.00001	99%	P <0.00001
aRR	15	1.17 (1.14–1.19)	P <0.00001	63%	P = 0.0005
**Age**					
<40 years	8	1.46 (1.17–1.82)	P = 0.0007	63%	P = 0.009
≥40 years	12	1.22 (1.10–1.35)	P = 0.0001	80%	P <0.00001
**Race**					
Caucasian	12	1.21 (1.16–1.27)	P <0.00001	64%	P = 0.001
Asian	5	1.10 (1.02–1.19)	P = 0.02	17%	P = 0.30
**Sex**					
Male	10	1.12 (1.06–1.19)	P <0.0001	31%	P = 0.16
Female	10	1.30 (1.18–1.42)	P <0.00001	68%	P = 0.0008
**Type of IBD and stroke**					
CD (IS)	2	1.23 (1.14–1.32)	P <0.00001	22%	P = 0.26
UC (IS)	2	1.14 (1.03–1.27)	P = 0.009	76%	P = 0.04
CD (HS)	2	1.38 (0.83–2.30)	P = 0.21	93%	P = 0.0001
UC (HS)	2	1.16 (0.85–1.58)	P = 0.35	87%	P = 0.006

CD, Crohn's disease; IBD, inflammatory bowel disease; UC, ulcerative colitis; HS, hemorrhagic stroke; IS, ischemic stroke; cRR, crude relative ratio; aRR, adjustment relative ratio; CI, confidence interval.

## 4 Discussion

Our research was aimed at synthesizing the best available evidence on the association between IBD and stroke risk. Compared with patients without IBD, our meta-analysis of 12 cohort studies revealed that patients with IBD have a significantly higher incidence rate of stroke (random-effects RR = 1.19, 95%CI 1.14–1.24; I^2^ = 59%). Our results remained consistent after subgroup analyses by race, sex, age, and year of follow-up, underscoring the robustness of our results. Furthermore, our results are also in line with previous research findings (20–23). To the best of our knowledge, our team conducted, for the first time, further research on the relationship between the IBD subtype and the risk of stroke subtype.

Of the five observational studies included in the previous meta-analysis, Singh et al. (23) reported that individuals with IBD were 18% more likely to suffer from stroke (adjusted OR, 1.18; 95%CI, 1.09–1.27). Moreover, the risk of stroke was higher in female patients (adjusted OR, 1.28; 95%CI, 1.17–1.41) compared to male patients (adjusted OR, 1.11; 95%CI, 0.98–1.25). Additionally, younger individuals (adjusted OR, 1.84; 95%CI, 1.28–2.66) were more likely to experience stroke than older patients (adjusted OR, 1.11; 95%CI, 1.02–1.21). This study only included four case-control studies and one cohort study. Case-control studies are more prone to recall bias, selection bias, and reverse causation, making them less reliable for establishing causality than cohort studies. Furthermore, their research only included Caucasians. Given these reasons, their conclusions need to be further verified. A meta-analysis that included eight articles (one case-control and seven cohorts) conducted by Xiao et al. (22) showed that, when compared to male, older, UC-type, and Caucasian IBD patients, female, younger, CD-type, and Asian patients had a higher risk of stroke. These results are basically consistent with our results and partially match the Yuan and Chen studies when it comes to race. However, Yuan et al. (21) conducted a subgroup analysis that revealed that the risk of stroke was higher in UC patients (adjusted RR, 1.32; 95%CI, 1.17–1.50) than in CD patients (adjusted RR, 1.17; 95%CI, 0.95–1.45), which is a contrast to our and Xiao's findings. The reason for this risk difference may have been observed by Ha et al. (42), who found no elevation of cerebrovascular occlusion risk in IBD patients (both UC and CD), however, this conclusion should be taken with caution. The authors suggested that cerebrovascular occlusion is the same as stroke, yet we held that a stroke must cause necrosis of brain tissue. Due to the presence of compensatory mechanisms in cerebral blood vessels, cerebrovascular occlusion does not always result in a stroke. The discrepancy between the findings of Yuan et al. and our study may be attributed to the varying sample sizes of stroke patients included in each study. Given these reasons, our conclusion may be more credible. All in all, compared with the article of Singh et al. (23), the other three articles (20–22) increased the number of cohort studies and expanded the scope of the population, making their conclusions more credible and increasing the scope of application of the conclusions.

However, compared with the previous four articles, our research has the largest number of cohort studies (*n* = 12) and the largest sample size (*n* = 4,495,055), especially by including newer cohort studies (26, 34–36, 43) published in 2021 and 2023, and a more thorough statistical analysis was conducted (e.g., preselected subgroup analyses of the types of IBD, sex, age, race/ethnicity, and duration of follow-up were conducted, and the Egger test was used to evaluate publication bias). Although the findings of our research are in line with the previously published meta-analyses on IBD and stroke, based on these advantages, our study provides relatively strong evidence that IBD is significantly associated with an increased risk of stroke.

Although the mechanisms underlying the link between IBD and stroke risk are not yet fully understood, some hypotheses exist. First, as is well known, IBD is a chronic non-specific intestinal inflammatory disease. Some studies found that C-reactive protein, adherent molecules, and inflammatory cytokines are overexpressed in patients with IBD (44–46); these chronic inflammatory factors contribute to vascular endothelial dysfunction, carotid intimal thickening, and abnormal carotid atherosclerosis, all of which eventually lead to stroke (47–49). Second, the destruction of the normal intestinal microenvironment may aggravate this kind of inflammatory reaction (50, 51); due to intestinal absorption dysfunction, insufficient absorption of vitamin B6 can lead to an increase in homocysteine, which can also promote the occurrence of stroke (52–54). Third, our study shows that the incidence of stroke in women is higher than that in men (women: RR = 1.30, 95%CI, 1.18–1.42; men: RR = 1.12, 95%CI, 1.06–1.19), which is contrary to our traditional view. The reason for this is that the level of C-reactive protein is higher in women than in men, and the oral contraceptive pill, a blood clotting agent, is used by young women with IBD (55).

Our results revealed considerable heterogeneity (random-effects RR = 1.19, 95%CI, 1.14–1.24; I^2^ = 59%), so we conducted subgroup analyses to identify the sources of this heterogeneity. However, after considering the IBD and stroke subtypes, age, follow-up time, sex, and race, we concluded that they were not the main sources of heterogeneity. In addition, to evaluate the stability of pooled results, we conducted a sensitivity analysis with the omission method (i.e., omitting one study at a time); the results of our meta-analysis for IBD and stroke were observed to be relatively stable by this method.

This study has some strengths and limitations that should be acknowledged. First, compared with previous meta-analyses on this topic, our research encompasses the newest and most extensive set of literature, and the estimated effect sizes were the same in all the included studies, which can minimize heterogeneity. Second, our team found that some articles (11, 29, 31, 33, 38–41) featured sample sizes from the same database, which could compromise the accuracy of the results. Therefore, we conducted a thorough analysis of the sources of the sample size included in the article and excluded any articles that utilized duplicate databases, thus ensuring the authenticity and credibility of our conclusions. Third, we performed an extensive literature search and applied strict inclusion/exclusion criteria and rigorous quality assessment. Fourth, most of the included studies were of good quality and provided high-quality evidence on the topic. Lastly, due to the results with apparent heterogeneity, we conducted subgroup analyses by age, sex, race, duration of follow-up, and type of IBD and stroke to examine potential sources of heterogeneity. All these features make the conclusion more credible and convincing. While it has several strengths, we also acknowledge that some limitations should be pointed out. First, these articles are from various medical register databases, which may lead to incomplete or inaccurate extraction of important information, due to the patients having different social environments, educational experiences, and cultural customs. This information may result in the overestimation or underestimation of the association between IBD and stroke risk. Second, risk factors of cerebrovascular disease include uncontrolled risk factors, such as race, age, sex, heredity, and climate change, and controllable risk factors such as hypertension, diabetes, smoking, drinking, and obesity. However, the studies we included did not uniformly adjust all confounding factors, which may make the results difficult to interpret or confound associations. Third, most of the articles included in the discussion highlighted the relationship between IBD subtypes and stroke risk, however, only two articles (35, 37) discussed the relationship between IBD subtypes and stroke subtypes. There is a need for more research to confirm the specific relationship between the IBD subtype and stroke subtype. Fourth, IBD is a typical inflammatory disease, and an inflammatory reaction plays an important role in the pathogenesis of stroke. However, these articles did not mention the severity and treatment of IBD. It is necessary to further examine the relationship between the severity and treatment of IBD and the occurrence of stroke. Finally, we included eight articles (61.5%) from Europe, featuring the largest sample size, three (23.07%) from Asia, featuring a medium sample size, and only two (15.38%) from North America, featuring the smallest sample size. Therefore, the conclusion of this article is more suitable for European and Asian populations. Whether these observed findings can be extrapolated to other regions needs further verification.

## 5 Conclusions

Our findings point to a link between IBD and an elevated risk of stroke. To lessen the risk of stroke, it is recommended that patients with IBD have a stroke risk evaluation, especially young female patients; assessing the risk of ischemic stroke is of particular importance. To gain a fuller understanding of the association between the two, further research is necessary that takes into account factors such as stroke subtype, IBD severity and treatment, region, and other potential confounders.

## Data availability statement

The original contributions presented in the study are included in the article/Supplementary material, further inquiries can be directed to the corresponding author.

## Author contributions

J-SF and MW performed the literature search, drafted the manuscript, and designed the systematic review. MW and B-cS screened the literature. NC, Q-BZ, and J-SF extracted data and assessed the quality. M-JH and YL analyzed and interpreted the data. MW, J-SF, and NC were responsible for the research design, data analysis, and manuscript revision. All authors gave their approval for the submission of the final manuscript.
