# Supplementary material for: Association between inflammatory bowel disease and risk of stroke: a systematic review and meta-analysis of cohort studies
Source: Front Neurol. 2023 Nov 17;14:1204727. doi: 10.3389/fneur.2023.1204727 (PMC10693426; doi:10.3389/fneur.2023.1204727)
Supplement: Supplementary file 1 [file Data_Sheet_1.pdf]

SUPPLEMENTARY MATERIAL

Table S1. Representatives search strings for PubMed. (From inception to November 2022)	
**Databases**	**Search strings**
**PubMed**	(Stroke OR Cerebrovascular Accident OR Brain Vascular Accident OR Cerebrovascular Stroke OR Cerebral Stroke OR Cerebrovascular Disorder OR Cerebrovascular Disease OR Intracranial Vascular Disease OR Brain Vascular Disorder OR Intracranial Vascular Disorders OR Cerebrovascular Occlusion OR Cerebral Infarction OR Cerebral Infarction OR Subcortical Infarction OR brain Infarction OR Cerebral hemorrhage OR Cerebrum Hemorrhage OR Cerebral Parenchymal Hemorrhage OR Intracerebral Hemorrhage OR Cerebral Hemorrhages OR Cerebral Brain Hemorrhage) AND (Crohn's disease OR Crohn's Enteritis OR Crohn* Disease OR Granulomatous Enteritis OR Granulomatous Colitis OR Ulcerative Colitis OR Idiopathic Proctocolitis OR Ulcerative Colitis)

**Table S2. Description of excluded studies at the stage of eligibility according to the PRISMA flow chart.**			
**No.**	**First author**	**Publication year**	**Reason for exclusion**
1.	Ha et a	2009	Studies were not cohort designed
2.	Yarur et a	2011	Studies were not cohort designed
3.	Osterman et al	2011	Studies were not cohort designed
4.	Sara et al	2020	Lack of the outcome of interest
5.	Sara et al	2021	Lack of the outcome of interest
6.	Petra et al	2022	Lack of the outcome of interest
7.	Lin et al	2015	Lack of the outcome of interest
8.	Rungoe et al	2014	Lack of the outcome of interest
9	Allison et al	2020	Lack of the outcome of interest
10	Ana et al	2015	Lack of the outcome of interest
11	Aristeidis et al	2014	Lack of the outcome of interest
12	Kuenzig et al	2022	Case-report review or meta-analysis
13	Chen et al	2020	Case-report review or meta-analysis
14	Siddharth et al	2014	Case-report review or meta-analysis
15	Min et al	2016	Case-report review or meta-analysis
16	Xiao et al	2015	Case-report review or meta-analysis

17	Dorn et al	2007	Case-report review or meta-analysis
18	Anuraag et al	2022	Case-report review or meta-analysis
19	Ryusuke et al	2022	Case-report review or meta-analysis
20	Daniele	2019	Case-report review or meta-analysis
21	Fu et al	2018	Case-report review or meta-analysis
22	Gandhi et al	2021	Case-report review or meta-analysis

References for the table S2

1. Ha C, Magowan S, Accortt NA, et al. Risk of arterial thrombotic events in inflammatory bowel disease. Am J Gastroenterol 2009; 104:1445–1451.
2. Yarur AJ, Deshpande AR, Pechman DM, et al. Inflammatory bowel disease is associated with an increased incidence of cardiovascular events. Am J Gastroenterol 2011; 106:741–747.
3. Osterman MT, Yang YX, Brensinger C, et al. No increased risk of myocardial infarction among patients with ulcerative colitis or Crohn's disease. Clin Gastroenterol Hepatol 2011;9: 875–880.
4 Ghoneim S, Shah A, Dhorepatil A, Butt MU, Waghray N. The Risk of Cerebrovascular Accidents in Inflammatory Bowel Disease in the United States: A Population-Based National Study. Clin Exp Gastroenterol. 2020 May 4;13:123-129. doi: 10.2147/CEG.S250182. PMID: 32440189; PMCID: PMC7210027.
5. Ghoneim S, Weissman S, Wang L, Aziz M, Atoot A, Sandhu D, Swaminath A, Feuerstein JD. Impact of inflammatory bowel disease on hospital outcomes in acute ischemic stroke: a nationwide cohort study. Int J Colorectal Dis. 2021 Aug;36(8):1759-1764. doi: 10.1007/s00384-021-03912-y. Epub 2021 Mar 17. PMID: 33733312.
6. Golovics PA, Verdon C, Wetwittayakhlang P, Filliter C, Gonczi L, Hahn GD, Wild GE, Afif W, Bitton A, Bessissow T, Brassard P, Lakatos PL. Increased Prevalence of Myocardial Infarction and Stable Stroke Proportions in Patients with Inflammatory Bowel Diseases in Quebec in 1996-2015. J Clin Med. 2022 Jan 28;11(3):686. doi: 10.3390/jcm11030686. PMID: 35160136; PMCID: PMC8837182.
7. Lin TY, Chen YG, Lin CL, Huang WS, Kao CH. Inflammatory Bowel Disease Increases the Risk of Peripheral Arterial Disease: A Nationwide Cohort Study. Medicine (Baltimore). 2015 Dec;94(52):e2381. doi: 10.1097/MD.0000000000002381. PMID: 26717386; PMCID: PMC5291627.
8. Rungoe C, Basit S, Ranthe MF, Wohlfahrt J, Langholz E, Jess T. Risk of ischaemic heart disease in patients with inflammatory bowel disease: a nationwide Danish cohort study. Gut. 2013 May;62(5):689-94. doi: 10.1136/gutjnl-2012-303285. Epub 2012 Sep 8. PMID: 22961677.
9. Bigeh A, Sanchez A, Maestas C, Gulati M. Inflammatory bowel disease and the risk for cardiovascular disease: Does all inflammation lead to heart disease? Trends Cardiovasc Med. 2020 Nov;30(8):463-469. doi: 10.1016/j.tcm.2019.10.001. Epub 2019 Oct 11. PMID: 31653485.
10. Filimon AM, Negreanu L, Doca M, Ciobanu A, Preda CM, Vinereanu D. Cardiovascular involvement in inflammatory bowel disease: Dangerous liaisons. World J Gastroenterol. 2015 Sep 7;21(33):9688-92. doi: 10.3748/wjg.v21.i33.9688. PMID: 26361415; PMCID: PMC4562952.
11. Katsanos AH, Kosmidou M, Giannopoulos S, Katsanos KH, Tsivgoulis G, Kyritsis AP, Tsianos EV. Cerebral arterial infarction in inflammatory bowel diseases. Eur J Intern Med. 2014 Jan;25(1):37-44. doi: 10.1016/j.ejim.2013.08.702. Epub 2013 Sep 9. PMID: 24028931.

12. Kuenzig ME, Fung SG, Marderfeld L, Mak JWY, Kaplan GG, Ng SC, Wilson DC, Cameron F, Henderson P, Kotze PG, Bhatti J, Fang V, Gerber S, Guay E, Kotteduwa Jayawarden S, Kadota L, Maldonado D F, Osei JA, Sandarage R, Stanton A, Wan M; InsightScope Pediatric IBD Epidemiology Group; Benchimol EI. Twenty-first Century Trends in the Global Epidemiology of Pediatric-Onset Inflammatory Bowel Disease: Systematic Review. Gastroenterology. 2022 Apr;162(4):1147- 1159.e4. doi: 10.1053/j.gastro.2021.12.282. Epub 2022 Jan 5. PMID: 34995526.

13. Chen Y, Wang X. Increased risk of stroke among patients with inflammatory bowel disease: A PRISMA-compliant meta-analysis. Brain Behav. 2021 Jun;11(6):e02159. doi: 10.1002/brb3.2159. Epub 2021 May 7. PMID: 33960728; PMCID: PMC8213927.

14. Singh S, Singh H, Loftus EV Jr, Pardi DS. Risk of cerebrovascular accidents and ischemic heart disease in patients with inflammatory bowel disease: a systematic review and meta-analysis. Clin Gastroenterol Hepatol. 2014 Mar;12(3):382-93.e1: quiz e22. doi: 10.1016/j.cgh.2013.08.023. Epub 2013 Aug 24. PMID: 23978350.

15. Yuan M, Zhou HY, Xiao XL, Wang ZQ, Yao-Zhi, Yin XP. Inflammatory bowel disease and risk of stroke: A meta-analysis of cohort studies. Int J Cardiol. 2016 Jan 1;202:106-9. doi: 10.1016/j.ijcard.2015.08.190. Epub 2015 Aug 29. PMID: 26386936.

16. Xiao Z, Pei Z, Yuan M, Li X, Chen S, Xu L. Risk of Stroke in Patients with Inflammatory Bowel Disease: A Systematic Review and Meta-analysis. J Stroke Cerebrovasc Dis. 2015 Dec;24(12):2774-80. Doi, 10.1016/j.jstrokecerebrovasdis.2015.08.008. Epub 2015 Sep 8. PMID: 26360973.

17 Dorn SD, Sandler RS. Inflammatory bowel disease is not a risk factor for cardiovascular disease mortality: results from a systematic review and meta-analysis. Am J Gastroenterol 2007; 102:662–667.

18. Jena A, James D, Singh AK, Dutta U, Sebastian S, Sharma V. Effectiveness and Durability of COVID-19 Vaccination in 9447 Patients With IBD: A Systematic Review and Meta-Analysis. Clin Gastroenterol Hepatol. 2022 Jul;20(7):1456-1479.e18. doi: 10.1016/j.cgh.2022.02.030. Epub 2022 Feb 19. PMID: 35189387; PMCID: PMC8856753.

19. Nambu R, Warner N, Mulder DJ, Kotlarz D, McGovern DPB, Cho J, Klein C, Snapper SB, Griffiths AM, Iwama I, Muise AM. A Systematic Review of Monogenic Inflammatory Bowel Disease. Clin Gastroenterol Hepatol. 2022 Apr;20(4):e653-e663. doi: 10.1016/j.cgh.2021.03.021. Epub 2021 Mar 18. PMID: 33746097; PMCID: PMC8448782.

20. Piovani D, Danese S, Peyrin-Biroulet L, Nikolopoulos GK, Lytras T, Bonovas S. Environmental Risk Factors for Inflammatory Bowel Diseases: An Umbrella Review of Meta-analyses. Gastroenterology. 2019 Sep;157(3):647-659.e4. doi: 10.1053/j.gastro.2019.04.016. Epub 2019 Apr 20. PMID: 31014995.

21. Fu Y, Lee CH, Chi CC. Association of Psoriasis with Inflammatory Bowel Disease: A Systematic Review and Meta-analysis. JAMA Dermatol. 2018 Dec 1;154(12):1417-1423. doi: 10.1001/jamadermatol.2018.3631. PMID: 30422277; PMCID: PMC6583370.

22. Gandhi A, Shah A, Jones MP, Koloski N, Talley NJ, Morrison M, Holtmann G. Methane positive small intestinal bacterial overgrowth in inflammatory bowel disease and irritable bowel syndrome: A systematic review and meta-analysis. Gut Microbes. 2021 Jan-Dec;13(1):1933313. doi: 10.1080/19490976.2021.1933313. PMID: 34190027; PMCID: PMC8253120.

Table S3 The Characteristics of included studies about continents, study subjects,, confounders adjustment and corresponding data in the meta-analysis				
**First author (year)**	**Continents**	**Study subjects**	**Confounders adjustment**	**RR/HR (95%CI)**
Alayo2022	Europe	the United Kingdom Biobank	Townsend Deprivation Index, traditional cardiovascular risk factors (body mass index, smoking status, average weekly alcohol intake, hypertension, diabetes, dyslipidemias at enrolment), physical activity, diet and C-reactive protein level at enrolment	cHR：<br>IBD:0.94(0.70-1.25)<br>UC:0.86(0.62-1.20)<br>CD:0.99(0.63-1.56)<br>aHR：<br>IBD:0.91(0.70-1.18)<br>UC:0.83(0.60-1.17)<br>CD: 0.98(0.62-1.55）
Baean-Diez 2018	Europe	The Catalan Institute of Health in Catalonia	age, sex, smoking status, total cholesterol, high density lipoprotein. cholesterol, systolic blood pressure and diastolic blood pressure. statins, hypertensive drugs and three categories of exposure to antirheumatic-specific treatments: disease-modifying antirheumatic drugs, other anti-inflammatory drugs, no exposure. CIID	aHR：<br>1.23(1.06 - 1.43)

Bernstein 2008	North America	Manitoba Health administrative database	NR	IRR：<br>IBD:1.16 (0.98–1.36)<br>CD: 1.32 (1.05–1.66)<br>UC: 1.03 (0.82–1.29)
Choi 2019	Asia	National Health Insurance Service (NHIS) of South Korea	age,sex,residencetype,income category hypertension,diabetes mellitus and dyslipidaemia.	aHR：<br>CD：1.18 (0.93-1.48)<br>UC: 1.05(0.95-1.16）
Christian 2021	Europe	the Disease Analyzer database (IQVIA)	NR	HR：<br>IBD: 1.30(1.06-1.59)<br>CD：1.50 (1.10-2.06)<br>UC: 1.17(0.90-1.52）
Huang 2014	Asia	the National Health Insurance Research Database (NHIRD)	Age, sex, comorbidities	aHR： IBD：<br>1.12(1.02-1.23)<br>CD:1.15(1.04-1.28)<br>UC:1.01(0.84-1.21)
Julien 2017	Europe	the French National Hospital Discharge Database	NR	cIR:<br>IBD:2.9(2.7-3.0)<br>SIR:<br>IBD:1.19(1.13-1.24)<br>CD:1.33(1.23-1.43)<br>UC:1.11(1.04-1.17)
Oh 2022	Asia	Korean National Health Insurance Service (NHIS)	NR	HR<br>CD:1.88(1.05-3.37)<br>UC:1.00(0.72-1.40)

Sun 2023	Europe	Epidemiology Strengthened by histoPathology Reports in Sweden（ESPRESSO）<br>Swedish National Patient Register (NPR)	birth year, sex, county of residence, and calendar year country of birth, educational attainment, number of healthcare visits, ischemic heart disease, heart failure, arrhythmias, hypertension, diabetes, obesity, dyslipidemia, chronic kidney disease, and COPD	aHR：<br>IBD： stroke:1.13 (1.08-1.17)<br>IS: 1.14 (1.09-1.18)<br>HS: 1.06 (0.97-1.15)<br>UC： stroke 1.15 (1.07-1.24)<br>IS: 1.19 (1.10-1.29)<br>HS: 1.07 (0.91-1.26)<br>CD： stroke 1.09 (1.04-1.15)<br>IS:1.09 (1.04-1.16)<br>HS: 1.00 (0.89-1.12)
Kristensen 2014	Europe	the National Patient Register	Age, calendar-year, gender, comorbidity, medical treatment,and socioeconomic status	aRR：<br>IBD:1.16(1.05-1.26)<br>CD:1.41(1.17-1.70)<br>UC: 1.10(0.99-1.22)
TimothyR 2021	Europe	the Clinical Practice Research Datalink (CPRD)<br>linked to Hospital Episode Statistics (HES) data and Office for National Statistics (ONS) mortality data.	age in years, gender, smoking status , diabetes , hypertension , dyslipidaemia , alcohol intake , 5ASA use , BMI , hospitalization	aHR:<br>IBD:1.10(0.99-1.24)
Zoller 2012	Europe	the National Board of Health and Welfare and Statistics Sweden.	age, period, socioeconomic status, region of residence, hospitalization of chronic lower respiratory diseases, obesity, alcoholism, hypertension,diabetes, atrial	SIR:<br>CD:IS 1.28(1.16-1.41)<br>HS 1.80(1.46-2.21)<br>UC:IS 1.21(1.12-1.31)<br>HS 1.37(1.13-1.66)

			fibrillation, heart failure, renal disease, sepsis, and coronary heart disease.	
aHR:adjust hazard ratios;cHR:crude hazard ratios;SIR:standardized incidence ratio;cIR:crude incidence ratio;aRR:adjust relative risk.IBD:inflammatory bowel disease;UC:ulcerative colitis;CD:Crohn’s disease;NR:no report;ASA:aminosalicylic acid;BMI:body mass index;IS:ischemic stroke;HS:hemorrhagic stroke.IS：Ischaemic stroke，HS：Hemorrhagic stroke,CIID:chronic immune-mediated inflammatory disease				

Table S4 Methodological quality assessment of included studies with NOS.

First author	Selection	Comparability	Exposure/Outcome	Total (0-9)	Quality
Alayo2022	✵✵	✵✵	✵✵	6	Moderate
Baean-Diez 2018	✵✵✵✵	✵✵	✵	7	High
Bernstein 2008	✵✵✵✵	✵✵	✵✵✵	9	High
Choi 2019	✵✵✵✵	✵✵	✵✵✵	9	High
Christian 2021	✵✵✵	✵	✵✵✵	7	High
Huang 2014	✵✵✵✵	✵✵	✵✵✵	9	High
Julien 2017	✵✵✵	✵	✵✵	6	Moderate
Oh 2022	✵✵✵	✵✵	✵✵✵	8	High
Sun2023	✵✵✵	✵✵	✵✵✵	8	High
Kristensen 2014	✵✵✵✵	✵	✵✵✵	8	High
TimothyR 2021	✵✵	✵✵	✵✵	6	Moderate
Zoller 2012	✵✵✵✵	✵✵	✵✵✵	9	High

Note: NOS, Newcastle–Ottawa Scale.Y: yes, N:no, U: unclear

Table S5 Results of sensitivity analyses			
**Studies omitted**	**RR (95% CI)**	***P*association**	**Heterogeneity**
Alayo 2022	1.19（1.14-1.24）	P<0.00001	*62%*
Baean-Diez 2018	1.19（1.14-1.24）	P<0.00001	*61%*
Bernstein 2008	1.19（1.14-1.25）	P<0.00001	*62%*
Choi (CD)2019	1.19（1.14-1.24）	P<0.00001	*62%*
Choi (UC)2019	1.20（1.15-1.25）	P<0.00001	*57%*
Christian 2021	1.19（1.14-1.24）	P<0.00001	*61%*
Huang 2014	1.20（1.14-1.25）	P<0.00001	*61%*
Julien 2017	1.19（1.14-1.26）	P<0.00001	61%
Kristensen 2014	1.19（1.14-1.25）	P<0.00001	62%
Oh(CD）2022	1.19（1.14-1.24）	P<0.00001	59%
Oh(UC）2022	1.19（1.14-1.24）	P<0.00001	61%
Sun2023	1.20（1.14-1.26）	P<0.00001	56%
Timothy R 2021	1.20（1.15-1.25）	P<0.00001	61%
Zoller (CD HS)2012	1.17（1.13-1.21）	P<0.00001	32%
Zoller (CD IS)2012	1.18（1.13-1.23）	P<0.00001	58%
Zoller (UC HS)2012	1.18（1.13-1.23）	P<0.00001	59%
Zoller (UC IS)2012	1.19（1.14-1.24）	P<0.00001	*61%*

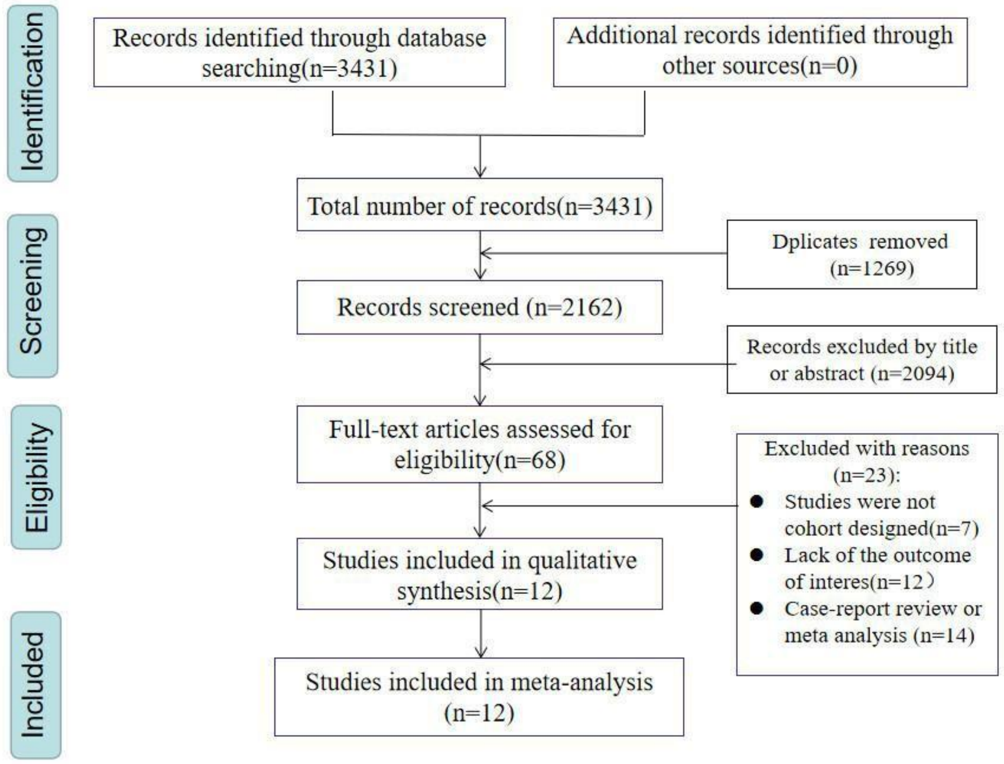

Figure S1. PRISMA (Preferred Reporting Items for Systematic Reviews and Meta-Analyses) flow diagram for studies included in and excluded from the meta-meta-analysis.

Study or Subgroup	log[Risk Ratio]	SE	Weight	Risk Ratio IV, Random, 95% CI
Alayo 2022	0.1681	0.1339	2.2%	1.18 [0.91, 1.54]
Baean-Diez 2018	0.207	0.0769	5.0%	1.23 [1.06, 1.43]
Bernstein 2008	0.1484	0.086	4.3%	1.16 [0.98, 1.37]
Choi(CD)2019	0.1655	0.1215	2.6%	1.18 [0.93, 1.50]
Choi(UC) 2019	0.0488	0.0511	7.8%	1.05 [0.95, 1.16]
Christian 2021	0.2624	0.1027	3.3%	1.30 [1.06, 1.59]
Huang 2014	0.1133	0.0477	8.3%	1.12 [1.02, 1.23]
Julien 2017	0.174	0.021	12.3%	1.19 [1.14, 1.24]
Kristensen 2014	0.1484	0.0422	9.1%	1.16 [1.07, 1.26]
Oh(CD)2022	0.6313	0.2978	0.5%	1.88 [1.05, 3.37]
Oh(UC)2022	0	0.1717	1.4%	1.00 [0.71, 1.40]
Sun2023	0.1222	0.0177	12.7%	1.13 [1.09, 1.17]
TimothyR 2021	0.0953	0.0611	6.5%	1.10 [0.98, 1.24]
Zoller(CD HS) 2012	0.5878	0.1047	3.2%	1.80 [1.47, 2.21]
Zoller(CD IS) 2012	0.2469	0.0494	8.0%	1.28 [1.16, 1.41]
Zoller(UC HS) 2012	0.3148	0.098	3.6%	1.37 [1.13, 1.66]
Zoller(UC IS) 2012	0.1906	0.0405	9.3%	1.21 [1.12, 1.31]
**Total (95% CI)**			**100.0%**	**1.19 [1.14, 1.24]**

Heterogeneity: $Tau^2 = 0.00$; $Chi^2 = 39.24$, df = 16 (P = 0.001); $I^2 = 59\%$
Test for overall effect: Z = 8.10 (P < 0.00001)

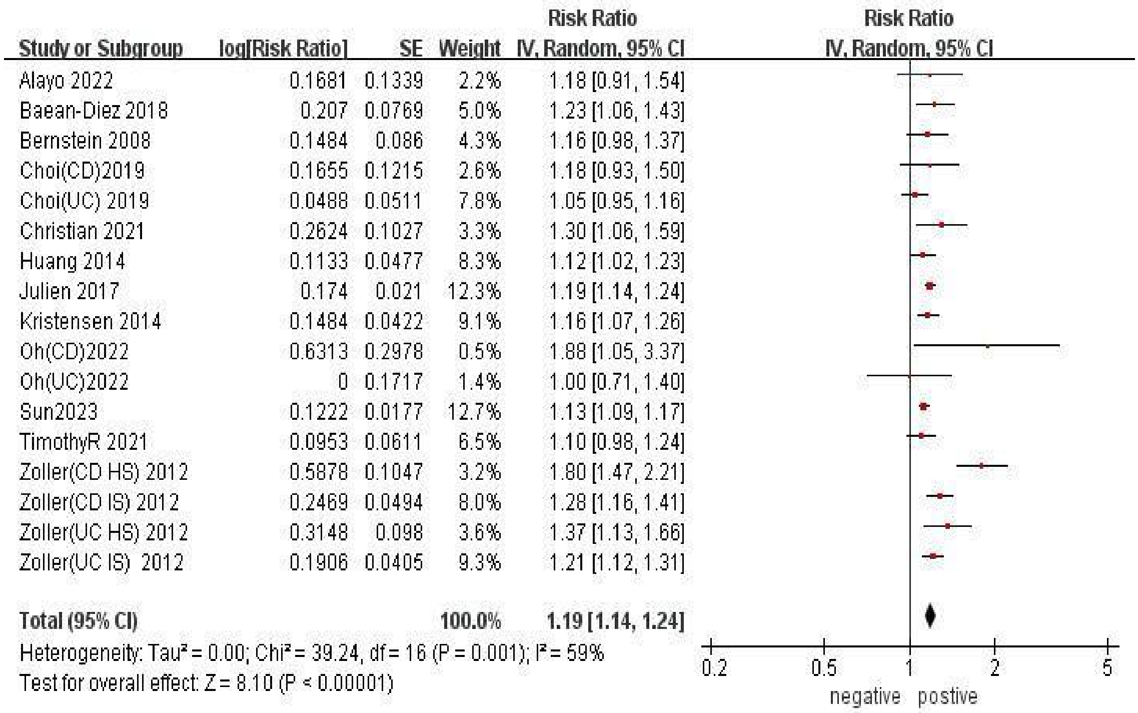

Figure S2. Meta-analysis of the association between IBD and stroke risk. The bottom row describes a combined overall effect which random-effects models were used to estimate.

Study or Subgroup	log[Risk Ratio]	SE	Weight	Risk Ratio IV, Random, 95% CI
**1.2.1 AnyIBD**				
TimothyR 2021	0.0953	0.0611	3.9%	1.10 [0.98, 1.24]
Huang 2014	0.1133	0.0477	4.8%	1.12 [1.02, 1.23]
Bernstein 2008	0.1484	0.086	2.7%	1.16 [0.98, 1.37]
Kristensen 2014	0.1484	0.0422	5.1%	1.16 [1.07, 1.26]
Alayo 2022	0.1681	0.1339	1.4%	1.18 [0.91, 1.54]
Julien 2017	0.174	0.021	6.6%	1.19 [1.14, 1.24]
Sun(IBD)2023	0.1906	0.0559	4.2%	1.21 [1.08, 1.35]
Baean-Diez 2018	0.207	0.0769	3.1%	1.23 [1.06, 1.43]
Christian 2021	0.2624	0.1027	2.1%	1.30 [1.06, 1.59]
**Subtotal (95% CI)**			**33.9%**	**1.18 [1.14, 1.21]**
Heterogeneity: Tau² = 0.00; Chi² = 4.25, df = 8 (P = 0.83); I² = 0%				
Test for overall effect: Z = 10.71 (P < 0.00001)				
**1.2.2 CD**				
Alayo 2022	-0.0202	0.2339	0.5%	0.98 [0.62, 1.55]
Huang 2014	0.1398	0.0546	4.3%	1.15 [1.03, 1.28]
Sun(CD)2023	0.1398	0.0384	5.4%	1.15 [1.07, 1.24]
Choi(CD)2019	0.1655	0.1215	1.7%	1.18 [0.93, 1.50]
Zoller(CD IS) 2012	0.2469	0.0494	4.6%	1.28 [1.16, 1.41]
Bernstein 2008	0.2776	0.1169	1.8%	1.32 [1.05, 1.66]
Julien 2017	0.2852	0.037	5.5%	1.33 [1.24, 1.43]
Kristensen 2014	0.3436	0.0954	2.4%	1.41 [1.17, 1.70]
Christian 2021	0.4055	0.1619	1.0%	1.50 [1.09, 2.06]
Zoller(CD HS) 2012	0.5878	0.1047	2.1%	1.80 [1.47, 2.21]
Oh(CD)2022	0.6313	0.2978	0.3%	1.88 [1.05, 3.37]
**Subtotal (95% CI)**			**29.6%**	**1.30 [1.20, 1.41]**
Heterogeneity: Tau² = 0.01; Chi² = 28.25, df = 10 (P = 0.002); I² = 65%				
Test for overall effect: Z = 6.48 (P < 0.00001)				
**1.2.3 UC**				
Alayo 2022	-0.1863	0.1752	0.9%	0.83 [0.59, 1.17]
Oh(UC)2022	0	0.1717	0.9%	1.00 [0.71, 1.40]
Huang 2014	0.01	0.0922	2.5%	1.01 [0.84, 1.21]
Bernstein 2008	0.0296	0.1148	1.8%	1.03 [0.82, 1.29]
Choi(UC) 2019	0.0488	0.0511	4.5%	1.05 [0.95, 1.16]
Sun(UC)2023	0.0862	0.0273	6.2%	1.09 [1.03, 1.15]
Kristensen 2014	0.0953	0.0528	4.4%	1.10 [0.99, 1.22]
Julien 2017	0.1044	0.0269	6.2%	1.11 [1.05, 1.17]
Christian 2021	0.157	0.1335	1.4%	1.17 [0.90, 1.52]
Zoller(UC IS) 2012	0.1906	0.0405	5.3%	1.21 [1.12, 1.31]
Zoller(UC HS) 2012	0.3148	0.098	2.3%	1.37 [1.13, 1.66]
**Subtotal (95% CI)**			**36.4%**	**1.11 [1.06, 1.16]**
Heterogeneity: Tau² = 0.00; Chi² = 15.55, df = 10 (P = 0.11); I² = 36%				
Test for overall effect: Z = 4.65 (P < 0.00001)				
**Total (95% CI)**			**100.0%**	**1.18 [1.14, 1.22]**
Heterogeneity: Tau² = 0.00; Chi² = 75.42, df = 30 (P < 0.00001); I² = 60%				
Test for overall effect: Z = 9.33 (P < 0.00001)				
Test for subgroup differences: Chi² = 12.61, df = 2 (P = 0.002), I² = 84.1%				

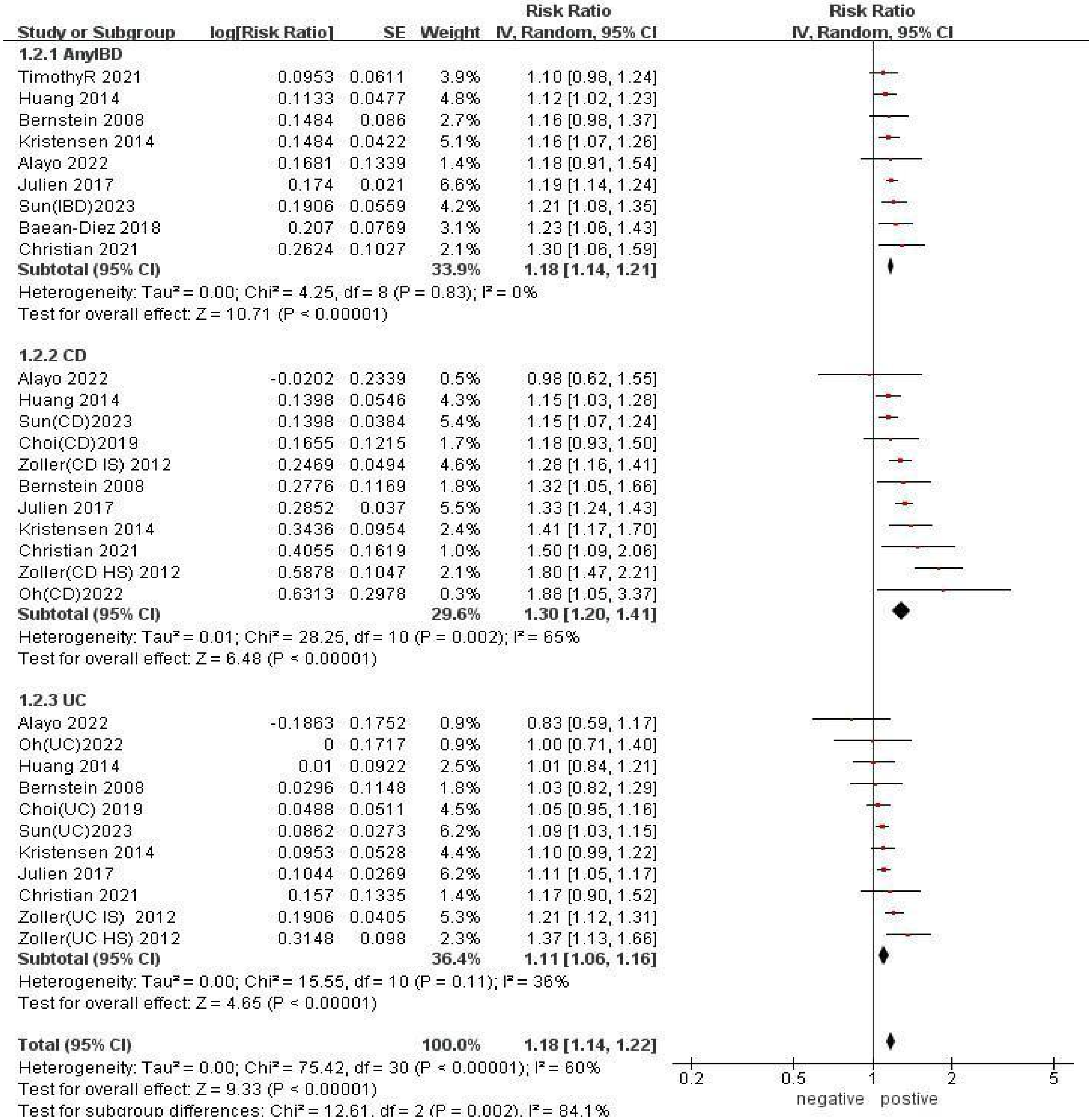

Figure S3. Meta-analysis of the association between the types of IBD and stroke risk. The bottom row of each type of IBD describes the comprehensive overall effect estimated using a random-effects model.

Study or Subgroup	log[Risk Ratio]	SE	Weight	Risk Ratio IV, Fixed, 95% CI
**1.7.1 <5year**				
Choi(CD)2019	0.1655	0.1215	0.8%	1.18 [0.93, 1.50]
Choi(UC) 2019	0.0488	0.0511	4.3%	1.05 [0.95, 1.16]
Christian 2021	0.2624	0.1027	1.1%	1.30 [1.06, 1.59]
Julien 2017	0.174	0.021	25.5%	1.19 [1.14, 1.24]
Zoller(CD HS) 2012	0.5878	0.1047	1.0%	1.80 [1.47, 2.21]
Zoller(CD IS) 2012	0.2469	0.0494	4.6%	1.28 [1.16, 1.41]
Zoller(UC HS) 2012	0.3148	0.098	1.2%	1.37 [1.13, 1.66]
Zoller(UC IS) 2012	0.1906	0.0405	6.9%	1.21 [1.12, 1.31]
**Subtotal (95% CI)**			**45.3%**	**1.21 [1.17, 1.24]**
Heterogeneity: Chi² = 26.10, df = 7 (P = 0.0005); I² = 73%				
Test for overall effect: Z = 11.87 (P < 0.00001)				
**1.7.2 ≥5year**				
Alayo 2022	0.1681	0.1339	0.6%	1.18 [0.91, 1.54]
Baean-Diez 2018	0.207	0.0769	1.9%	1.23 [1.06, 1.43]
Bernstein 2008	0.1484	0.086	1.5%	1.16 [0.98, 1.37]
Huang 2014	0.1133	0.0477	4.9%	1.12 [1.02, 1.23]
Kristensen 2014	0.1484	0.0422	6.3%	1.16 [1.07, 1.26]
Oh(CD)2022	0.6313	0.2978	0.1%	1.88 [1.05, 3.37]
Oh(UC)2022	0	0.1717	0.4%	1.00 [0.71, 1.40]
Sun2023	0.1222	0.0177	35.9%	1.13 [1.09, 1.17]
TimothyR 2021	0.0953	0.0611	3.0%	1.10 [0.98, 1.24]
**Subtotal (95% CI)**			**54.7%**	**1.14 [1.10, 1.17]**
Heterogeneity: Chi² = 5.33, df = 8 (P = 0.72); I² = 0%				
Test for overall effect: Z = 8.89 (P < 0.00001)				
**Total (95% CI)**			**100.0%**	**1.17 [1.14, 1.19]**
Heterogeneity: Chi² = 39.24, df = 16 (P = 0.001); I² = 59%				
Test for overall effect: Z = 14.56 (P < 0.00001)				
Test for subgroup differences: Chi² = 7.80, df = 1 (P = 0.005), I² = 87.2%				

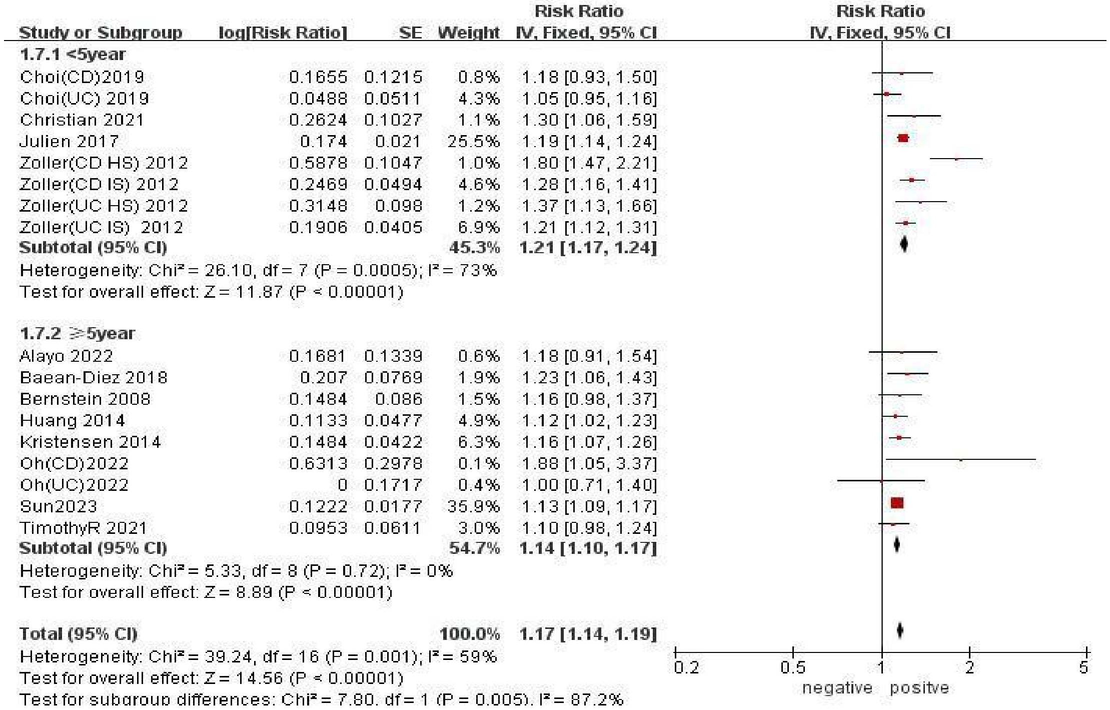

Figure S4. Meta-analysis of the association between different follow-up periods of IBD and stroke risk. The bottom row of each follow-up period describes the comprehensive overall effect estimated using a random-effects model.

Study or Subgroup	log[Risk Ratio]	SE	Weight	Risk Ratio IV, Random, 95% CI
**1.5.1 <40year**				
Bernstein 2008	0.4253	0.7603	0.3%	1.53 [0.34, 6.79]
Choi(CD)2019	0.6981	0.3016	1.8%	2.01 [1.11, 3.63]
Choi(UC) 2019	0.6206	0.2353	2.6%	1.86 [1.17, 2.95]
Christian 2021	0.8587	0.5185	0.7%	2.36 [0.85, 6.52]
Huang 2014	-0.0726	0.1385	4.8%	0.93 [0.71, 1.22]
Julien 2017	0.2231	0.1447	4.6%	1.25 [0.94, 1.66]
Sun(18) 2023	0.8544	0.2204	2.9%	2.35 [1.53, 3.62]
Sun(<40) 2023	0.27	0.0623	7.4%	1.31 [1.16, 1.48]
**Subtotal (95% CI)**			**25.3%**	**1.46 [1.17, 1.82]**
Heterogeneity: Tau² = 0.05; Chi² = 18.75, df = 7 (P = 0.009); I² = 63%				
Test for overall effect: Z = 3.39 (P = 0.0007)				
**1.5.2 ≥40year**				
Bernstein 2008	0.4187	0.2288	2.7%	1.52 [0.97, 2.38]
Bernstein2008(60)	0.0953	0.0891	6.5%	1.10 [0.92, 1.31]
Choi(CD)2019	0.077	0.1287	5.1%	1.08 [0.84, 1.39]
Choi(UC) 2019	0.0198	0.0567	7.6%	1.02 [0.91, 1.14]
Christian 2021(40)	0.4318	0.1679	4.0%	1.54 [1.11, 2.14]
Christian2021(60)	0.131	0.1332	5.0%	1.14 [0.88, 1.48]
Huang 2014(50)	0.207	0.0875	6.5%	1.23 [1.04, 1.46]
Huang2014(65)	0.0862	0.0658	7.3%	1.09 [0.96, 1.24]
Julien 2017	0.5481	0.0663	7.3%	1.73 [1.52, 1.97]
Julien2017(55)	0.3221	0.0626	7.4%	1.38 [1.22, 1.56]
Julien2017(75)	0.0677	0.0669	7.3%	1.07 [0.94, 1.22]
Kristensen 2014	0.1484	0.0422	8.0%	1.16 [1.07, 1.26]
**Subtotal (95% CI)**			**74.7%**	**1.22 [1.10, 1.35]**
Heterogeneity: Tau² = 0.02; Chi² = 54.48, df = 11 (P < 0.00001); I² = 80%				
Test for overall effect: Z = 3.83 (P = 0.0001)				
**Total (95% CI)**			**100.0%**	**1.27 [1.16, 1.38]**
Heterogeneity: Tau² = 0.02; Chi² = 76.84, df = 19 (P < 0.00001); I² = 75%				
Test for overall effect: Z = 5.14 (P < 0.00001)				
Test for subgroup differences: Chi² = 2.23, df = 1 (P = 0.14), I² = 55.2%				

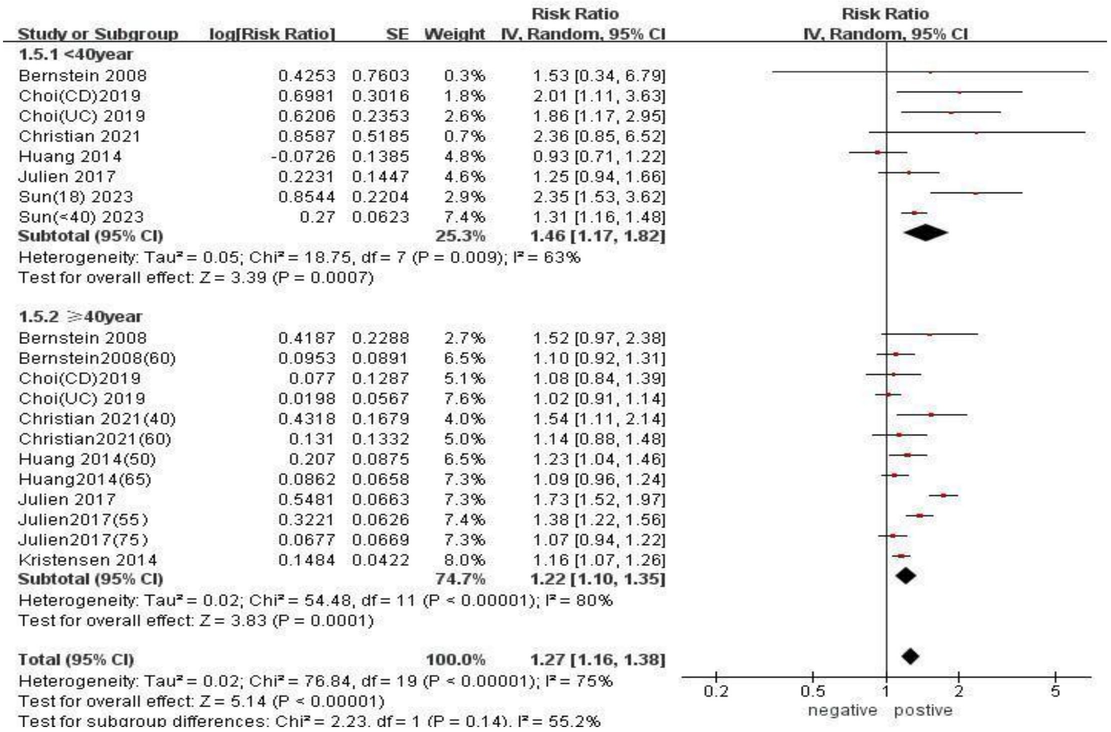

Figure S5. Meta-analysis of the association between different age groups and the risk of stroke in individuals with IBD. The bottom row of each age group describes the comprehensive overall effect estimated using a random-effects model.

Study or Subgroup	log[Risk Ratio]	SE	Weight	Risk Ratio IV, Random, 95% CI
**1.4.1 male**				
Bernstein 2008	0.0953	0.1267	3.5%	1.10 [0.86, 1.41]
Choi(CD)2019	0.0677	0.1655	2.4%	1.07 [0.77, 1.48]
Choi(UC) 2019	0.01	0.0706	6.3%	1.01 [0.88, 1.16]
Christian 2021	0.1222	0.1445	3.0%	1.13 [0.85, 1.50]
Huang 2014	0.1222	0.0676	6.4%	1.13 [0.99, 1.29]
Sun2023	0.0583	0.0281	8.9%	1.06 [1.00, 1.12]
Zoller(CD HS) 2012	0.4574	0.1476	2.9%	1.58 [1.18, 2.11]
Zoller(CD IS) 2012	0.1655	0.0762	5.9%	1.18 [1.02, 1.37]
Zoller(UC HS) 2012	0.1906	0.1296	3.4%	1.21 [0.94, 1.56]
Zoller(UC IS) 2012	0.174	0.0529	7.4%	1.19 [1.07, 1.32]
**Subtotal (95% CI)**			**50.1%**	**1.12 [1.06, 1.19]**
Heterogeneity: Tau² = 0.00; Chi² = 13.03, df = 9 (P = 0.16); I² = 31%				
Test for overall effect: Z = 3.95 (P < 0.0001)				
**1.4.2 Female**				
Bernstein 2008	0.1823	0.1104	4.2%	1.20 [0.97, 1.49]
Choi(CD)2019	0.27	0.165	2.4%	1.31 [0.95, 1.81]
Choi(UC) 2019	0.0862	0.078	5.8%	1.09 [0.94, 1.27]
Christian 2021	0.4187	0.1476	2.9%	1.52 [1.14, 2.03]
Huang 2014	0.0953	0.0693	6.3%	1.10 [0.96, 1.26]
Sun2023	0.1823	0.0289	8.8%	1.20 [1.13, 1.27]
Zoller(CD HS) 2012	0.7324	0.1425	3.0%	2.08 [1.57, 2.75]
Zoller(CD IS) 2012	0.3148	0.0663	6.5%	1.37 [1.20, 1.56]
Zoller(UC HS) 2012	0.5008	0.1421	3.0%	1.65 [1.25, 2.18]
Zoller(UC IS) 2012	0.2231	0.0615	6.8%	1.25 [1.11, 1.41]
**Subtotal (95% CI)**			**49.9%**	**1.30 [1.18, 1.42]**
Heterogeneity: Tau² = 0.01; Chi² = 28.54, df = 9 (P = 0.0008); I² = 68%				
Test for overall effect: Z = 5.63 (P < 0.00001)				
**Total (95% CI)**			**100.0%**	**1.21 [1.14, 1.28]**
Heterogeneity: Tau² = 0.01; Chi² = 56.75, df = 19 (P < 0.0001); I² = 67%				
Test for overall effect: Z = 6.39 (P < 0.00001)				
Test for subgroup differences: Chi² = 6.75, df = 1 (P = 0.009), I² = 85.2%				

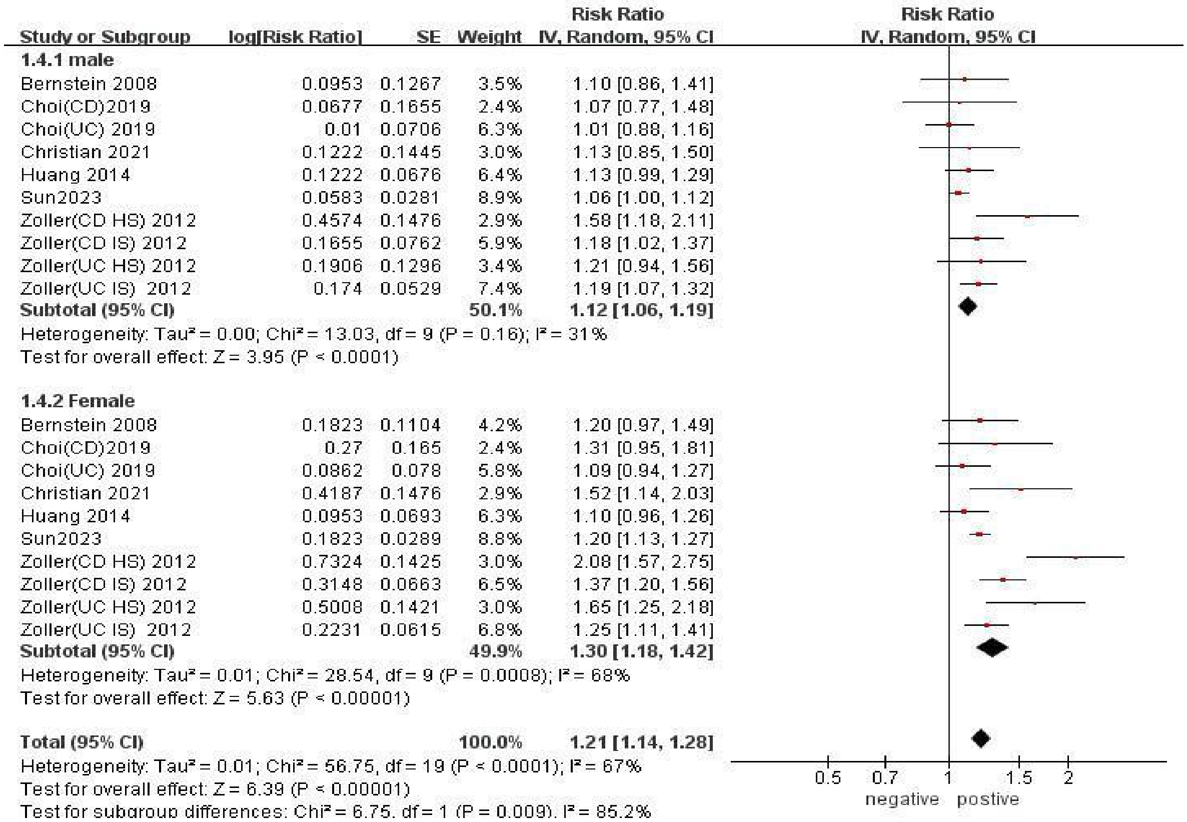

Figure S6. Meta-analysis of the association between different gender groups and the risk of stroke in individuals with IBD. The bottom row of each gender group describes the comprehensive overall effect estimated using a random-effects model.

Study or Subgroup	log[Risk Ratio]	SE	Weight	Risk Ratio IV, Random, 95% CI
**1.3.1 Caucasian**				
Alayo 2022	0.1681	0.1339	2.2%	1.18 [0.91, 1.54]
Baean-Diez 2018	0.207	0.0769	5.0%	1.23 [1.06, 1.43]
Bernstein 2008	0.1484	0.086	4.3%	1.16 [0.98, 1.37]
Christian 2021	0.2624	0.1027	3.3%	1.30 [1.06, 1.59]
Julien 2017	0.174	0.021	12.3%	1.19 [1.14, 1.24]
Kristensen 2014	0.1484	0.0422	9.1%	1.16 [1.07, 1.26]
Sun2023	0.1222	0.0177	12.7%	1.13 [1.09, 1.17]
TimothyR 2021	0.0953	0.0611	6.5%	1.10 [0.98, 1.24]
Zoller(CD HS) 2012	0.5878	0.1047	3.2%	1.80 [1.47, 2.21]
Zoller(CD IS) 2012	0.2469	0.0494	8.0%	1.28 [1.16, 1.41]
Zoller(UC HS) 2012	0.3148	0.098	3.6%	1.37 [1.13, 1.66]
Zoller(UC IS) 2012	0.1906	0.0405	9.3%	1.21 [1.12, 1.31]
**Subtotal (95% CI)**			**79.5%**	**1.21 [1.16, 1.27]**
Heterogeneity: $Tau^2 = 0.00$; $Chi^2 = 30.43$, df = 11 (P = 0.001); $I^2 = 64\%$				
Test for overall effect: Z = 8.03 (P < 0.00001)				
**1.3.2 Asian**				
Choi(CD)2019	0.1655	0.1215	2.6%	1.18 [0.93, 1.50]
Choi(UC) 2019	0.0488	0.0511	7.8%	1.05 [0.95, 1.16]
Huang 2014	0.1133	0.0477	8.3%	1.12 [1.02, 1.23]
Oh(CD)2022	0.6313	0.2978	0.5%	1.88 [1.05, 3.37]
Oh(UC)2022	0	0.1717	1.4%	1.00 [0.71, 1.40]
**Subtotal (95% CI)**			**20.5%**	**1.10 [1.02, 1.19]**
Heterogeneity: $Tau^2 = 0.00$; $Chi^2 = 4.85$, df = 4 (P = 0.30); $I^2 = 17\%$				
Test for overall effect: Z = 2.39 (P = 0.02)				
**Total (95% CI)**			**100.0%**	**1.19 [1.14, 1.24]**
Heterogeneity: $Tau^2 = 0.00$; $Chi^2 = 39.24$, df = 16 (P = 0.001); $I^2 = 59\%$				
Test for overall effect: Z = 8.10 (P < 0.00001)				
Test for subgroup differences: $Chi^2 = 4.22$, df = 1 (P = 0.04), $I^2 = 76.3\%$				

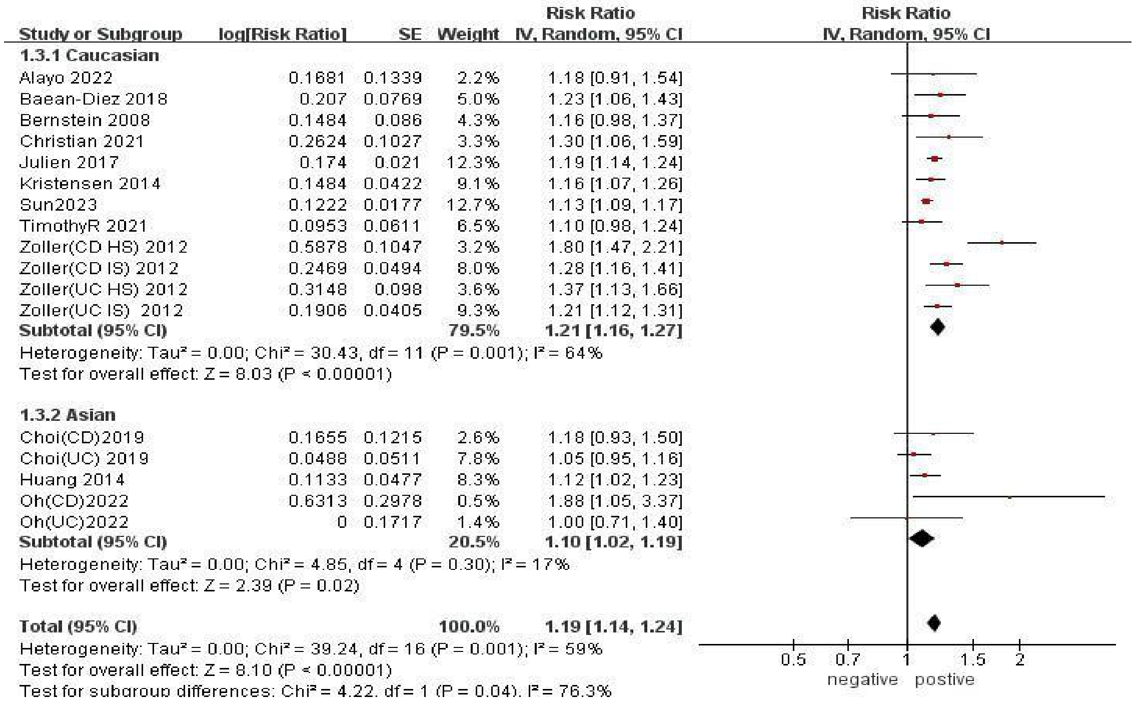

Figure S7.Meta-analysis of the association between different race groups of IBD and stroke risk. The bottom row of each region group describes the comprehensive overall effect estimated using a random-effects model.

Study or Subgroup	log[Risk Ratio]	SE	Weight	Risk Ratio IV, Fixed, 95% CI
**1.6.1 cRR**				
Alayo 2022	-0.0619	0.1454	0.4%	0.94 [0.71, 1.25]
Bernstein 2008	0.1484	0.0812	1.2%	1.16 [0.99, 1.36]
Christian 2021	0.2624	0.1027	0.8%	1.30 [1.06, 1.59]
Julien 2017	1.0647	0.0173	27.2%	2.90 [2.80, 3.00]
**Subtotal (95% CI)**			**29.6%**	**2.69 [2.61, 2.78]**
Heterogeneity: Chi² = 228.60, df = 3 (P < 0.00001); I² = 99%				
Test for overall effect: Z = 59.74 (P < 0.00001)				
**1.6.2 aRR**				
Alayo 2022	0.1681	0.1339	0.5%	1.18 [0.91, 1.54]
Baean-Diez 2018	0.207	0.0769	1.4%	1.23 [1.06, 1.43]
Choi(CD)2019	0.1655	0.1215	0.6%	1.18 [0.93, 1.50]
Choi(UC) 2019	0.0488	0.0511	3.1%	1.05 [0.95, 1.16]
Huang 2014	0.1133	0.0477	3.6%	1.12 [1.02, 1.23]
Julien 2017	0.174	0.021	18.4%	1.19 [1.14, 1.24]
Kristensen 2014	0.1484	0.0422	4.6%	1.16 [1.07, 1.26]
Oh(CD)2022	0.6313	0.2978	0.1%	1.88 [1.05, 3.37]
Oh(UC)2022	0	0.1717	0.3%	1.00 [0.71, 1.40]
Sun2023	0.1222	0.0177	26.0%	1.13 [1.09, 1.17]
TimothyR 2021	0.0953	0.0611	2.2%	1.10 [0.98, 1.24]
Zoller(CD HS) 2012	0.5878	0.1047	0.7%	1.80 [1.47, 2.21]
Zoller(CD IS) 2012	0.2469	0.0494	3.3%	1.28 [1.16, 1.41]
Zoller(UC HS) 2012	0.3148	0.098	0.8%	1.37 [1.13, 1.66]
Zoller(UC IS) 2012	0.1906	0.0405	5.0%	1.21 [1.12, 1.31]
**Subtotal (95% CI)**			**70.4%**	**1.17 [1.14, 1.19]**
Heterogeneity: Chi² = 38.12, df = 14 (P = 0.0005); I² = 63%				
Test for overall effect: Z = 14.27 (P < 0.00001)				
**Total (95% CI)**			**100.0%**	**1.49 [1.47, 1.52]**
Heterogeneity: Chi² = 2063.02, df = 18 (P < 0.00001); I² = 99%				
Test for overall effect: Z = 44.46 (P < 0.00001)				
Test for subgroup differences: Chi² = 1796.30, df = 1 (P < 0.00001), I² = 99.9%				

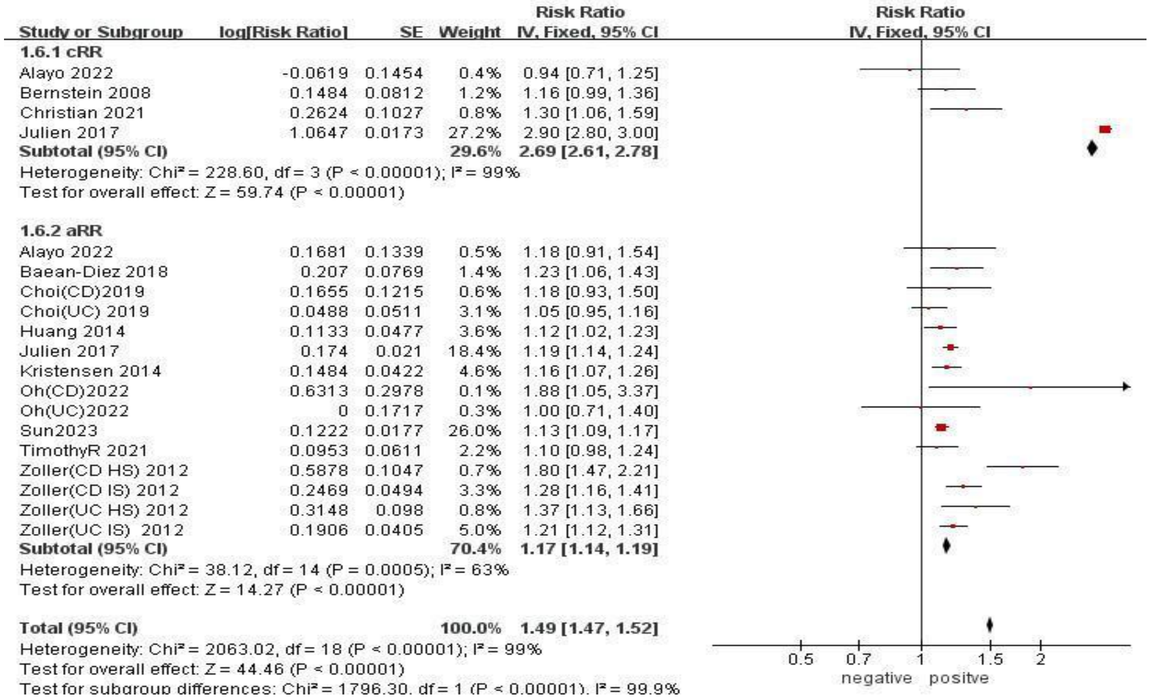

Figure S8. Meta-analysis of the association between whether to adjust confounding factors of IBD and stroke risk. The bottom row of each group describes the comprehensive overall effect estimated using a random-effects model.

Study or Subgroup	log[Risk Ratio]	SE	Weight	Risk Ratio IV, Random, 95% CI
**1.8.1 CD （IS）**				
Sun(CD IS) 2023	0.174	0.0412	14.7%	1.19 [1.10, 1.29]
Zoller(CD IS) 2012	0.2469	0.0494	13.9%	1.28 [1.16, 1.41]
**Subtotal (95% CI)**			**28.5%**	**1.23 [1.14, 1.32]**
Heterogeneity: Tau² = 0.00; Chi² = 1.28, df = 1 (P = 0.26); I² = 22%				
Test for overall effect: Z = 5.69 (P < 0.00001)				
**1.8.2 UC （IS）**				
Sun(UC IS) 2023	0.0862	0.0318	15.5%	1.09 [1.02, 1.16]
Zoller(UC IS) 2012	0.1906	0.0405	14.7%	1.21 [1.12, 1.31]
**Subtotal (95% CI)**			**30.2%**	**1.14 [1.03, 1.27]**
Heterogeneity: Tau² = 0.00; Chi² = 4.11, df = 1 (P = 0.04); I² = 76%				
Test for overall effect: Z = 2.60 (P = 0.009)				
**1.8.3 CD （HS）**				
Sun(CD HS) 2023	0.0677	0.0834	10.5%	1.07 [0.91, 1.26]
Zoller(CD HS) 2012	0.5878	0.1047	8.6%	1.80 [1.47, 2.21]
**Subtotal (95% CI)**			**19.1%**	**1.38 [0.83, 2.30]**
Heterogeneity: Tau² = 0.13; Chi² = 15.10, df = 1 (P = 0.0001); I² = 93%				
Test for overall effect: Z = 1.25 (P = 0.21)				
**1.8.4 UC （HS）**				
Sun(UC HS) 2023	0	0.0578	13.0%	1.00 [0.89, 1.12]
Zoller(UC HS) 2012	0.3148	0.098	9.2%	1.37 [1.13, 1.66]
**Subtotal (95% CI)**			**22.2%**	**1.16 [0.85, 1.58]**
Heterogeneity: Tau² = 0.04; Chi² = 7.66, df = 1 (P = 0.006); I² = 87%				
Test for overall effect: Z = 0.94 (P = 0.35)				
**Total (95% CI)**			**100.0%**	**1.21 [1.11, 1.32]**
Heterogeneity: Tau² = 0.01; Chi² = 37.16, df = 7 (P < 0.00001); I² = 81%				
Test for overall effect: Z = 4.27 (P < 0.0001)				
Test for subgroup differences: Chi² = 1.56, df = 3 (P = 0.67), I² = 0%				

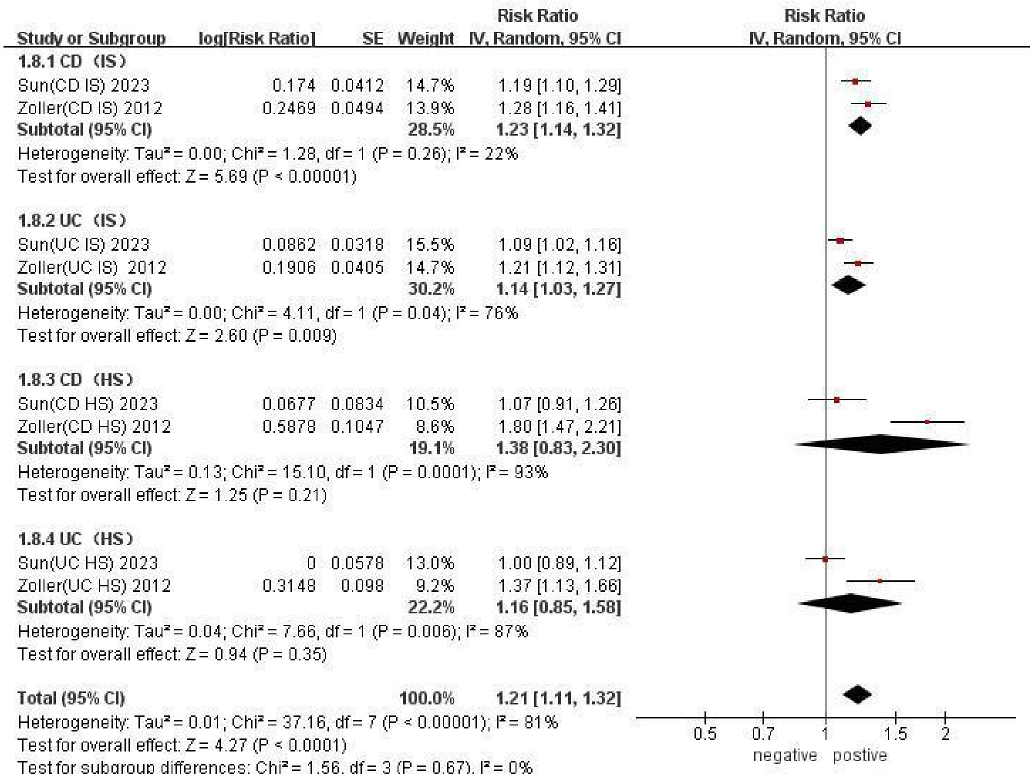

Figure S9. Meta-analysis of the association between IBD type and risk of stroke type. The bottom row of each type of IBD describes the comprehensive overall effect estimated using a random-effects model.

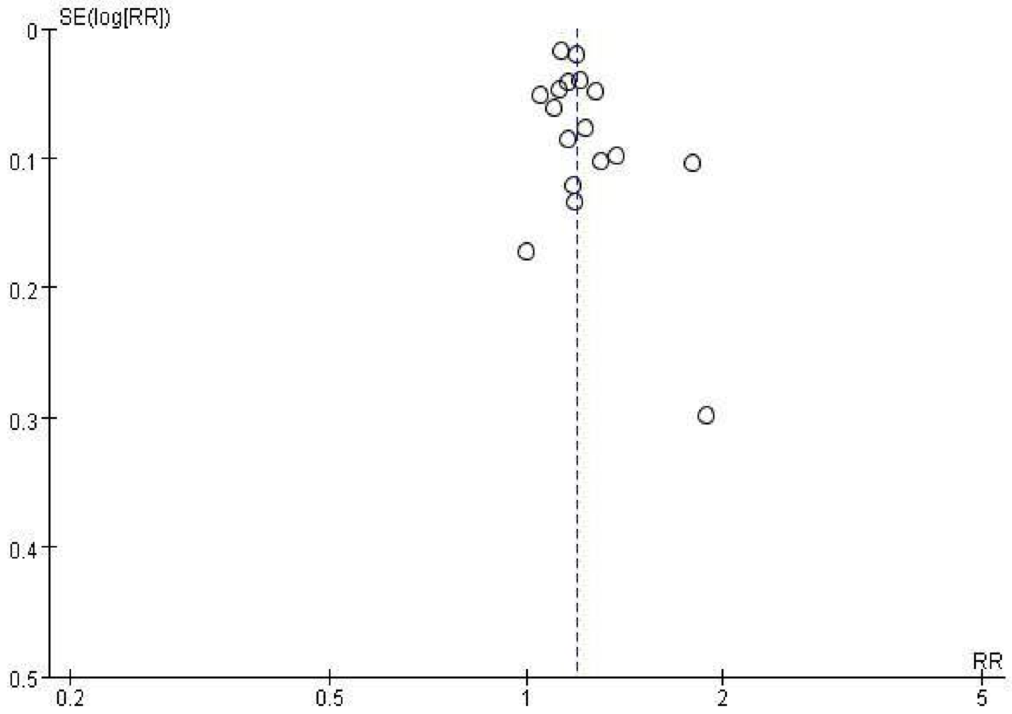

Figure S10. Funnel plot to detect risk of publication bias in the meta-analysis

Egger's test

Std_Eff	Coef.	Std. Err.	t	P>|t|	[95% Conf.	Interval]
slope	.1203383	.0259271	4.64	0.000	.0650759	.1756007
bias	.9819937	.5930324	1.66	0.119	-.2820249	2.246012

Figure S11. The Egger's test to detect risk of publication bias in the meta-analysis

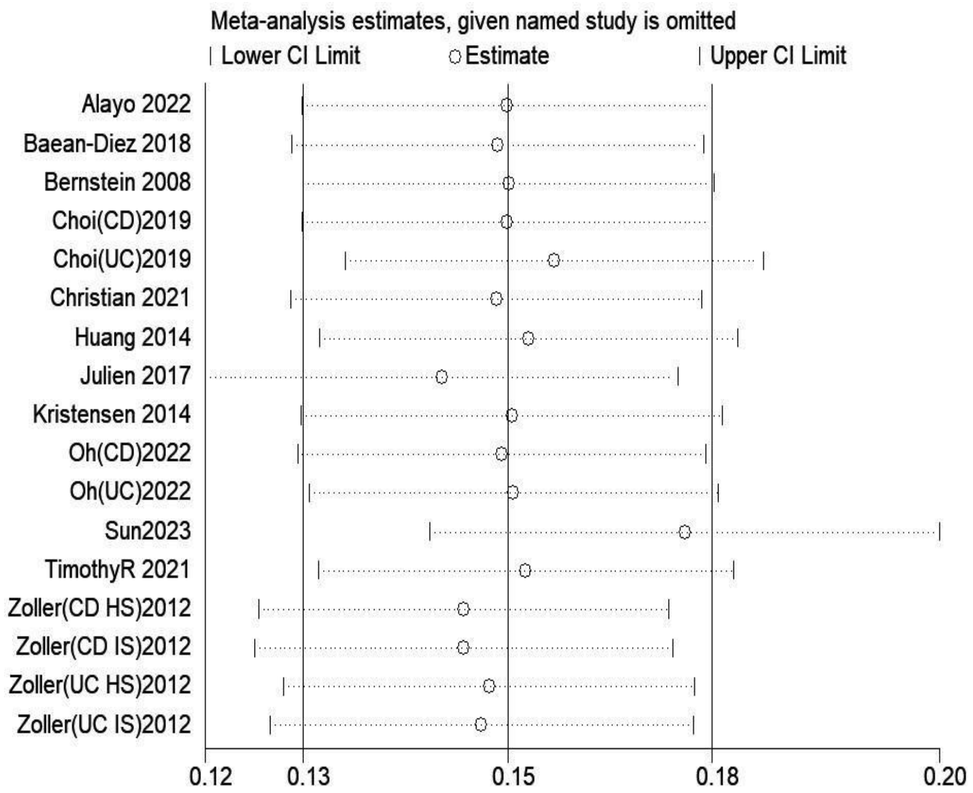

Figure S12. One-by-one elimination method to detect the sensitivity analysis in the meta-analysis
